# Resistance to cephalosporins and quinolones in *Escherichia coli* isolated from irrigation water from the Rímac river in east Lima, Peru

**DOI:** 10.17843/rpmesp.2024.412.13246

**Published:** 2024-06-13

**Authors:** Mónica Huamán Iturrizaga, Gina Salvador-Luján, Liliana Morales, Jeanne Alba Luna, Lino Velasquez Garcia, Julio Daniel Pacheco Perez, Maria J. Pons

**Affiliations:** 1 Microbial Ecology Laboratory, Faculty of Biological Sciences, Universidad Nacional Mayor de San Marcos, Lima, Peru. Microbial Ecology Laboratory Faculty of Biological Sciences Universidad Nacional Mayor de San Marcos Lima Peru; 2 Office of Epidemiology and Environmental Health, National Maternal Perinatal Institute, Lima Peru. Office of Epidemiology and Environmental Health National Maternal Perinatal Institute Lima Peru; 3 Microbiology Laboratory, “Luis Arias Schereiber” Central Military Hospital, Lima, Peru. Microbiology Laboratory “Luis Arias Schereiber” Central Military Hospital Lima Peru; 4 Microbiology Laboratory Guillermo Almenara Irigoyen National Hospital, Lima, Peru. Microbiology Laboratory Guillermo Almenara Irigoyen National Hospital Lima Peru; 5 Laboratory of Molecular Genetics and Biochemistry. Universidad Científica del Sur, Lima, Peru. Laboratory of Molecular Genetics and Biochemistry Universidad Científica del Sur Lima Peru

**Keywords:** Escherichia coli, antibiotic resistance, irrigation water, ESBL-producers, diarrhoeagenic E. coli.

## Abstract

**Objectives.:**

To evaluate the presence and sensitivity to antimicrobials of *Escherichia coli* strains isolated from 24 irrigation water samples from the Rimac river of East Lima, Peru.

**Materials and methods.:**

The *E. coli* strains were identified by PCR. Antibiotic susceptibility was processed by the disk diffusion method. Genes involved in extended spectrum beta-lactamases (BLEE), quinolones and virulence were determined by PCR.

**Results.:**

All samples exceeded the acceptable limits established in the Environmental Quality Standards for vegetable irrigation. Of the 94 isolates, 72.3% showed resistance to at least one antibiotic, 24.5% were multidrug resistant (MDR) and 2.1% were extremely resistant. The highest percentages of resistance were observed for ampicillin-sulbactam (57.1%), nalidixic acid (50%), trimethoprim-sulfamethoxazole (35.5%) and ciprofloxacin (20.4%). Among the isolates, 3.2% had a BLEE phenotype related to the *bla*_CTX-M-15_ gene. *qnrB* (20.4%) was the most frequent transferable mechanism of resistance to quinolones, and 2.04% had *qnrS*. It was estimated that 5.3% were diarrheagenic *E. coli* and of these, 60% were enterotoxigenic *E. coli*, 20% were enteropathogenic *E. coli* and 20% were enteroaggregative *E. coli*.

**Conclusions.:**

The results show the existence of diarrheogenic pathotypes in the water used for irrigation of fresh produce and highlight the presence of BLEE- and MDR-producing *E. coli*, demonstrating the role played by irrigation water in the dissemination of resistance genes in Peru.

## INTRODUCTION

The emergence of antibiotic-resistant bacteria is a threat to global public health, which needs a multidisciplinary approach to integrate knowledge about “One Health”, including the environment, humans, and animals ^1^. Aquatic systems have been identified as important reservoirs of resistance ^2^^,^^3^, providing dissemination and transmission routes for antimicrobial-resistant bacteria to transfer to humans and animals ^4^.

The spread of antibiotic resistance in aquatic systems deserves special attention considering that water use can facilitate the transmission of bacteria to humans (e.g., in oral use, irrigation, recreation, and/or fishing) ^5^^-^^7^. Likewise, the presence of other compounds in these environments, such as metals and/or disinfectants, has been related to the co-selection or selection of resistance, which accumulates in contaminated aquatic systems ^8^^,^^9^. In addition, the origin of some of the most widespread antibiotic resistance genes associated with human infections (e.g., *bla*_CTX -M_) has been identified in aquatic environmental bacteria ^10^.

The Rimac River is the most important river basin in Peru. It is estimated that 15% of its water resources are used in agriculture, being the main source of water for agriculture in eastern Lima ^11^. The indicators of fecal contamination in this river exceed the category limits for vegetable irrigation (1000 most probable number [MPN]/100 ml) established by the Water Quality Standards of the Peruvian Ministry of the Environment ^11^.

It should be noted that not only pathogenic microorganisms are relevant for the mobilization of resistance mechanisms, but also commensal bacteria such as *Escherichia coli*, which is considered one of the most representative species of the gut microbiota in both humans and animals ^12^. The presence of *E. coli* is used as an indicator of water and food quality and some pathotypes are virulence factors associated with diarrhea (diarrheogenic *E. coli*).

The cephalosporins and quinolones are among the most widely used antimicrobials in humans and production animals. The extended-spectrum beta-lactamases (ESBL) are one of the most important resistance mechanisms in health and are widely distributed in the community; they confer resistance to beta-lactam antibiotics, as well as the mechanisms associated with resistance to aminoglycosides and quinolones, mainly due to chromosomal mutations, in addition to transferable mechanisms ^13^^,^^14^.

A better understanding of antibiotic resistance in specific irrigation systems is essential to create mitigation strategies in agriculture. In Peru, information on resistance levels in bacteria isolated from water bodies is limited, particularly regarding irrigation water ^12^. Therefore, this study aims to determine the levels of antibiotic resistance and to carry out the molecular characterization of ESBL and transferable mechanisms of quinolone resistance in *E. coli* isolated from irrigation water in eastern Lima, Peru.

**KEY MESSAGES**

Motivation for the study. Aquatic systems, including irrigation water, have been identified as reservoirs of antimicrobial resistance, with few studies in Peru on the presence of *Escherichia coli* and their levels of virulence and antimicrobial resistance.

Main findings. Our results show the presence of *E. coli* above the established standard for vegetable irrigation water, some with very high levels of antimicrobial resistance.

Implications. The presence of ESBL-producing strains of extended-spectrum beta-lactamases and multidrug-resistant *E. coli* in irrigation water could contribute to the dissemination of resistance genes in Peru, posing a significant threat to public health.

## MATERIALS AND METHODS

### Study area

**Figure 1:**
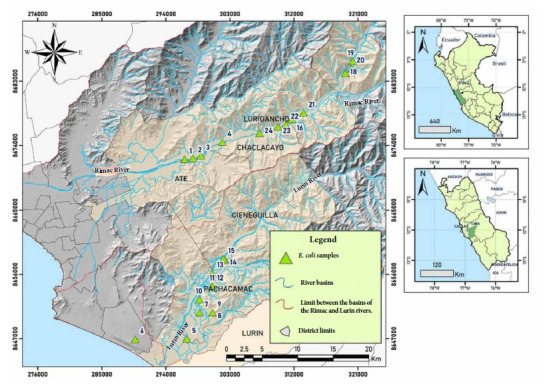
Map with irrigation water sampling points in the east of Lima, Peru.

We conducted a cross-sectional observational study. Water samples were collected from 24 irrigation water sampling points in 5 vegetable-growing areas from the districts of Lurigancho, Chaclacayo, Pachacamac, La Molina and Lurin, located on the eastern bank of the Rimac River in eastern Lima Peru, between October 2019 and February 2020 (Figure 1). The samples were obtained from the largest agricultural fields, particularly from irrigation canals entering these large parcels mainly with produce and short-stemmed vegetables. We should mention that no drinking water sources were found nearby nor was there any indication of the presence of sheep, cattle or other livestock.

This study was approved by the Vice-rector for Research and Postgraduate Studies of the Universidad Nacional Mayor de San Marcos under code B19101681.

### Isolation and identification of *Escherichia coli*

Samples were collected following protocol RD160-2015-DIGESA and transported to the Microbial Ecology laboratory of the Faculty of Biological Sciences of the Universidad Nacional Mayor de San Marcos ^15^. Samples were processed using the Colilert-18/Quanti-Tray method to analyze total coliforms and *E. coli* in all types of water (ISO 9308-2:2012). Strains phenotypically suspected to be *E. coli* were selected and stored on trypticase soy agar (TSA) at 8 °C for antibiotic susceptibility testing and at -80 °C in skim milk for molecular testing performed at the Molecular Genetics Laboratory of the Universidad Científica del Sur.

E. coli was molecularly identified by amplification of the *uidA* gene. DNA extraction was performed by heat shock for 5 min at 100°C, followed by centrifugation at 13,000 rpm for 5 min. The extracted DNA was stored at -20 °C until use. We used the 652-bp uidA-R primers CCA TCA GCA CGT TAT CGA ATC CTT 61 82.6µM uidA-F from a 652-bp amplicon to amplify the *uidA* gene, which encodes the 3-glucuronidase enzyme as a target for *E. coli* detection ^16^.

### Antimicrobial susceptibility

Confirmed *E. coli* strains were reactivated on TSA agar and antibiogram was performed with the Kirby Bauer disk diffusion method against 17 antibiotics: nalidixic acid (30 µg), trimethoprim sulfamethoxazole (1. 25/75 µg), ciprofloxacin (5 µg), ampicillin-sulbactam (10/10 µg), cefepime (30 µg), amoxicillin-clavulanic acid (20/10 µg), levofloxacin (5 µg), gentamicin (10 µg), fosfomycin (200 µg), aztreonam (30 µg), cefotaxime (30 µg), cefazolin (30 µg), cefoxitin (30 µg), ceftazidime (30 µg), imipenem (10 µg), meropenem (10 µg) and amikacin (30 µg). Inhibition halos were interpreted following the CLSI 2019 guideline ^38^. The Jarlier method was used for phenotypic detection of ESBL. The double-disk synergy method was used for AmpC-type beta-lactamases, and carbapenemase screening was considered with imipenem and meropenem inhibition halos < 22 mm. The control strains were *E. coli* ATCC 25922 and ATCC 35218. To obtain an overview, strains with intermediate and resistant inhibition halos were included in the resistant category. Multidrug resistance (MDR) was defined as the absence of acquired sensitivity to at least one agent in three or more antimicrobial categories, extreme resistance (XDR) was defined as resistance to three or more antimicrobial families, including carbapenemics ^1^^,^^17^^,^^18^.

### Diarrheogenic pathotype of *Escherichia coli*

Eight virulence genes associated with diarrheogenic *E. coli* (DEC) genes were detected by multiplex PCR: enterotoxigenic (lt, st), enteropathogenic (eaeA), Shiga toxin-producing (stx1, stx2), enteroinvasive (ipaH), enteroaggregative (aggR) and diffusely adherent (daaD) *E. coli*^19^.

### ESBL and transferable quinolone resistance mechanisms

Molecular characterization of *E. coli* ESBL genes was carried out in strains presenting the ESBL phenotype. Amplification was performed by PCR for *bla*_CTX -M_, *bla*_TEM_ and *bla*_SHV_^20^. The presence of *qnrA*, *qnrB*, *qnrC*, *qnrD*, *qnrVC*, *qnrS*, *qepA*, and *oqxAB* genes was determined by PCR in strains with decreased susceptibility (resistance or intermediate) to nalidixic acid ^14^.

## RESULTS

### Isolation and identification of *Escherichia coli*

Total fecal coliform and *E. coli* counts exceeded 2400 NMP/100 ml in the 24 processed water samples. We isolated 118 suspected E. coli colonies from among the Colilert-positive samples; of these, 95 (79.2%) were confirmed by molecular identification of the *uidA* gene.

### Antimicrobial Susceptibility

**Figure 2:**
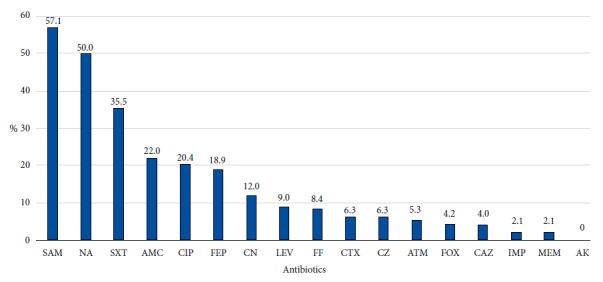
Levels of resistance to antibiotics in Escherichia coli isolated from irrigation water in Eastern Lima 2019-2020.

Significant levels of resistance to ampicillin sulbactam (57.1%), nalidixic acid (50.0%), trimethoprim-sulfamethoxazole (35.5%), amoxicillin-clavulanic acid (22.0%) and ciprofloxacin (20.4%) were found. With regard to beta-lactams, we found that resistance to cefepime was 18.9%, followed by cefazolin and cefotaxime with 6.3% resistance. The only antibiotic with no resistance was amikacin (0%) (Figure 2).

Antimicrobial susceptibility testing found that 68 (72.3%) strains were resistant to at least one antibiotic. Notably, 23 (24.5%) strains were MDR and 2 (2.1%) were XDR. Finally, 26 (27.6%) strains were sensitive to all antibiotics. (Supplementary material).

### Determination of the diarrheogenic pathotype in *Escherichia coli*

Of the 94 *E. coli* isolates, 5 (5.3%) had at least one diarrheogenic virulence gene, and of these, 3 (60%) had the enterotoxigenic virulence gene (*lt*, *st*) being ETEC, 1 (20.0%) enteropathogenic (*eaeA*) EPEC and 1 (20.0%) enteroaggregative (*aggR*) EAEC.

### Detection of ESBL and AmpC

Regarding beta-lactam resistance mechanisms, AmpC-type beta-lactamases were detected phenotypically in 5 (5.3%) strains and the ESBL phenotype in 3 (3.2%) strains. In addition, all ESBL -producing strains had the *bla*_CTX-M-15_ gene.

### Transferable mechanisms of quinolone resistance (TMQR)

Of the 49 strains with decreased sensitivity to quinolones, 10 strains had the *qnrB* gene (20.4%), being the most frequent, and only one strain (2.0%) had the *qnrS* gene.

## DISCUSSION

The presence of *E. coli* in aquatic environments has been related to the discharge of wastewater, whether from domestic or industrial use, releasing antibiotic-resistant bacteria into the environment ^21^. It should be noted that samples were obtained from agricultural waters that were directly exposed to the environment, so the contamination could have had different origins, such as the introduction of sewage, domestic discharges, and household and/or wild animal excrement ^21^^,^^22^.

The number of total coliforms and *E. coli* in the 24 irrigation water sampling points exceeded the permissible limit established by national regulations (DS N°004-2017-MINAM for vegetable irrigation) ^23^, in agreement with previous reports from national agencies ^24^. The poor quality of water used for irrigation is one of the reasons for the presence of pathogens in short-stemmed vegetables, which can be contaminated at any stage of the food chain, from planting to the consumer ^25^. In addition, contamination of irrigation water and the prevalence of *E. coli* in vegetables affects human health ^26^.

In addition, diarrheogenic *E. coli* strains were detected (5.3%). These strains are characterized by their capacity to cause pathologies in animals and humans through the transmission of foodborne diseases, thus compromising the use of water for vegetable irrigation. This percentage is lower in comparison with other countries in the region, such as Chile, which reported 10% of diarrheogenic *E. coli* strains in surface water used for vegetable irrigation using a tangential filtration method, ^27^^)^ which tends to concentrate the bacterial load, and 14% found in irrigation water in Sinaloa, Mexico. Our findings show that ETEC was the most frequently isolated pathotype, although EPEC and EAEC have also been reported. Previous studies of diarrheogenic *E. coli* in a cohort of children in Lima have described these pathotypes, with ETEC being more frequent in children 2 to 12 months of age with diarrhea ^28^. High levels of resistance to sulfonamides were found in these strains, and also to quinolones which are not commonly used in this age group ^28^.

The presence of antibiotic resistance and the respective genes involved is generally linked to anthropogenic effect, such as human feces and sewage ^21^ and is also related to a high load of contaminants (heavy metals, antibiotics and pesticides) in waters, mainly due to industry activities (mining), population growth and agricultural activities ^29^. It is important to note that, in our study, the sampled area had no hospital or industrial waste channels ^29^.

In recent years, increased levels of antimicrobial resistance in irrigation water have been reported in Texas ^30^ and also in the Latin American region ^31^. Thus, in the present study, 72.3% of *E. coli* strains were resistant to at least one antibiotic, 24.5% were MDR, and 2.1% were XDR. In fact, resistance levels are extremely high in most river systems (up to 98% of the total bacteria detected), followed by lakes, with lower values reported in ponds and springs (<1%) ^32^.

The most frequent resistance phenotypes in this study were related to quinolones and sulfonamides. The presence of genes related to sulfonamide resistance has been associated with wastewater effluents (neither chlorinated nor dechlorinated) ^33^. On the other hand, resistance to quinolones has increased in relation to the therapeutic use and growth promoters, which has been increasing worldwide ^34^. High levels of quinolone resistance have been reported in microorganisms isolated from both diarrheic and healthy children ^35^^-^^36^ in Peru, as in other areas of the region, indicating the high pressure of this antibiotic in the population.

Aquatic environments have been considered important reservoirs regarding the transferable mechanisms involved in quinolone resistance ^37^. Water genetic studies have reported the presence of the *qepA* and *aac (6')-Ib-cr* genes, encoding fluoroquinolone resistance, in a high percentage of sewage and sludge samples ^38^. Our results show that the *qnrB* gene (20.4%) was the most frequent transferable mechanism of quinolone resistance (TMQR), and only one isolate had the *qnrS* gene. A correlation was previously established between the presence of a TMQR, such as *qepA* and *qnrS*, and the amount of Cu and Zn in vegetative soils with long-term manure application, correlating heavy metals with the persistence of antibiotic resistance genes ^39^.

Our findings of ESBL strains (3.2%) were lower compared with the reports by Palacios (16.1%) in water from the Piura River, Peru ^34^, and the 29% ESBL found in *E. coli* from irrigation water from Ecuador ^40^. However, these results are not comparable because, in these studies, they were selected with media containing antibiotics of the cephalosporin family. Likewise, the *bla*_CTX-M-15_ gene was detected, which together with the *bla*_CTX-M-55_, *bla*_CTX-M-65_ genes were the genes most frequently found in association with the ESBL phenotype, and were also the most frequent alleles associated with human infections ^3^^,^^14^^,^^41^.

The study presents some limitations related to the size of the samples, considering the large extension of the area. In addition, screening culture media were not used for the detection of ESBL strains, which would have helped to isolate a greater number of *E. coli* resistant to this group of antimicrobials. Nonetheless, the importance of this study lies in the contribution of our findings to the scarce existing information on *E. coli* in irrigation waters with resistance to different families of antibiotics (quinolones, aminoglycosides, beta-lactams, monobactams, sulfonamides). Likewise, the finding of *E. coli* pathotypes highlights the need to improve irrigation water policies and control in the different agricultural areas of Lima. This is particularly important in Peru, which has a low frequency of wastewater treatment.

In conclusion, this study showed the presence of fecal coliforms above the permissible limit established by the national standard. In addition, it demonstrates the existence of diarrheagenic *E. coli* and high levels of resistance to quinolones and sulfonamides, with special concern to ESBL-producing *E. coli*, in irrigation water from the periphery of Lima, representing a potential danger to animal and human health.

## References

[1] Pons MJ, de Toro M, Medina S, Sáenz Y, Ruiz-Blázquez J (2020). Antimicrobials, antibacterial resistance and sustainable health. South Sustainability.

[2] Marti E, Variatza E, Balcazar JL (2014). The role of aquatic ecosystems as reservoirs of antibiotic resistance. Trends Microbiol.

[3] Freitas DY, Araújo S, Folador ARC, Ramos RTJ, Azevedo JSN, Tacão M (2019). Extended Spectrum Beta-Lactamase-Producing Gram-Negative Bacteria Recovered From an Amazonian Lake Near the City of Belém, Brazil. Front Microbiol.

[4] Larsson DGJ, Flach CF (2022). Antibiotic resistance in the environment. Nat Rev Microbiol.

[5] Duarte AC, Rodrigues S, Afonso A, Nogueira A, Coutinho P (2022). Antibiotic Resistance in the Drinking Water: Old and New Strategies to Remove Antibiotics, Resistant Bacteria, and Resistance Genes. Pharmaceuticals (Basel).

[6] Blaustein RA, Shelton DR, Van Kessel JA, Karns JS, Stocker MD, Pachepsky YA (2016). Irrigation waters and pipe-based biofilms as sources for antibiotic-resistant bacteria. Environ Monit Assess.

[7] Li Y, Zhang C, Mou X, Zhang P, Liang J, Wang Z (2022). Distribution characteristics of antibiotic resistance bacteria and related genes in urban recreational lakes replenished by different supplementary water source. Water Sci Technol.

[8] Jiao YN, Chen H, Gao RX, Zhu YG, Rensing C (2017). Organic compounds stimulate horizontal transfer of antibiotic resistance genes in mixed wastewater treatment systems. Chemosphere.

[9] Gupta S, Graham DW, Sreekrishnan TR, Ahammad SZ (2022). Effects of heavy metals pollution on the co-selection of metal and antibiotic resistance in urban rivers in UK and India. Environ Pollut.

[10] Poirel L, Kämpfer P, Nordmann P (2002). Chromosome-encoded Ambler class A beta-lactamase of Kluyvera georgiana, a probable progenitor of a subgroup of CTX-M extended-spectrum beta-lactamases. Antimicrob Agents Chemother.

[11] MdDAy Riego (2012). Resultado del monitoreo de la calidad del agua en la cuenca del río Rímac: Informe Técnico.

[12] Castillo AK, Espinoza K, Chaves AF, Guibert F, Ruiz J, Pons MJ (2022). Antibiotic susceptibility among non-clinical Escherichia coli as a marker of antibiotic pressure in Peru (2009-2019): one health approach. Heliyon.

[13] Foudraine DE, Strepis N, Stingl C, Ten Kate MT, Verbon A, Klaassen CHW (2021). Exploring antimicrobial resistance to beta-lactams, aminoglycosides and fluoroquinolones in E. coli and K. pneumoniae using proteogenomics. Sci Rep.

[14] Palma N, Pons MJ, Gomes C, Mateu J, Riveros M, García W (2017). Resistance to quinolones, cephalosporins and macrolides in Escherichia coli causing bacteraemia in Peruvian children. J Glob Antimicrob Resist.

[15] DIGESA (2016). Protocolo para la toma de muestra. Resolución Directoral.

[16] Bej AK, DiCesare JL, Haff L, Atlas RM (1991). Detection of Escherichia coli and Shigella spp in water by using the polymerase chain reaction and gene probes for uid. Appl Environ Microbiol.

[17] Clinical and Laboratory Standards Institute Performance Standards for Antimicrobial Susceptibility Testing (2021). CLSI supplement M100 (ISBN 978-1-68440-105-5.

[18] Magiorakos AP, Srinivasan A, Carey RB, Carmeli Y, Falagas ME, Giske CG (2012). Multidrug-resistant, extensively drug-resistant and pandrug-resistant bacteria an international expert proposal for interim standard definitions for acquired resistance. Clin Microbiol Infect.

[19] Guion CE, Ochoa TJ, Walker CM, Barletta F, Cleary TG (2008). Detection of diarrheagenic Escherichia coli by use of melting-curve analysis and real-time multiplex PCR. J Clin Microbiol.

[20] Pons MJ, Vubil D, Guiral E et al (2015). Characterisation of extended-spectrum ß-lactamases among Klebsiella pneumoniae isolates causing bacteraemia and urinary tract infection in Mozambique. J Glob Antimicrob Resist.

[21] Karkman A, Pärnänen K, Larsson DGJ (2019). Fecal pollution can explain antibiotic resistance gene abundances in anthropogenically impacted environment. Nat Commun.

[22] Vega-Sánchez V, Talavera-Rojas M, Barba-León J, Zepeda-Velázquez AP, Reyes-Rodríguez NE (2020). La resistencia antimicrobiana en Escherichia coli aislada de canales y heces bovinas de rastros en el centro de México. Rev Mex Cienc Pecu.

[23] Md Ambiente (2017). Aprueban Estándares de Calidad Ambiental (ECA) para Agua y Establecen Disposiciones Complementarias. DS N° 004-2017-MINAM.

[24] MdDAy Riego (2012). Resultado del monitoreo de la calidad del agua en la cuenca del río Rímac: Informe técnico. Informe Técnico.

[25] Graczyk Z, Graczyk TK, Naprawska A (2011). A role of some food arthropods as vectors of human enteric infections. Cent Eur J Biol.

[26] Escobedo C, Ariza E (2014). Nivel de contaminación fecal en hortalizas expedidas en mercados de Huanuco y su relación en el riego con aguas residuales no tratadas. Investigación Valdizana.

[27] Rojas-Aedo J, Morales O, Jara M, Morales O, Martínez MC (2013). Conference: Sociedad de Microbiología de Chile.

[28] Ochoa TJ, Ecker L, Barletta F, Mispireta ML, Gil AI, Contreras C (2009). Age-related susceptibility to infection with diarrheagenic Escherichia coli among infants from Periurban areas in Lima, Peru. Clin Infect Dis.

[29] Deblonde T, Cossu-Leguille C, Hartemann P (2011). Emerging pollutants in wastewater a review of the literature. Int J Hyg Environ Health.

[30] Duffy EA, Lucia LM, Kells JM, Castillo A, Pillai SD, Acuff GR (2005). Concentrations of Escherichia coli and genetic diversity and antibiotic resistance profiling of Salmonella isolated from irrigation water, packing shed equipment, and fresh produce in Texas. J Food Prot.

[31] Díaz-Gavidia C, Barría C, Weller DL, Salgado-Caxito M, Estrada EM, Araya A (2022). Humans and Hoofed Livestock Are the Main Sources of Fecal Contamination of Rivers Used for Crop Irrigation: A Microbial Source Tracking Approach. Front Microbiol.

[32] Nnadozie CF, Odume ON (2019). Freshwater environments as reservoirs of antibiotic resistant bacteria and their role in the dissemination of antibiotic resistance genes. Environ Pollut.

[33] Fahrenfeld N, Ma Y, O'Brien M, Pruden A (2013). Reclaimed water as a reservoir of antibiotic resistance genes: distribution system and irrigation implications. Front Microbiol.

[34] Riaz L, Mahmood T, Khalid A, Rashid A, Ahmed-Siddique MB, Kamal A (2018). Fluoroquinolones in the environment A review on their abundance, sorption and toxicity in soil. Chemosphere.

[35] Pons M, Mosquito S, Gomes C, Del Valle LJ, Ochoa TJ, Ruiz J (2014). Analysis of quinolone-resistance in commensal and diarrheagenic Escherichia coli isolates from infants in Lima, Peru. Trana R Soc Trop Med Hyg.

[36] Pons M, Mosquito S, Ochoa T (2012). Niveles de Resistencia a antimicrobianos, en especial a quinolonas, en cepas de Escherichia coli comensales en niños de la zona periurbana de Lima, Perú. Rev Peru Med Exp Salud Pública.

[37] Miranda CD, Concha C, Godoy FA, Lee MR (2022). Aquatic Environments as Hotspots of Transferable Low-Level Quinolone Resistance and Their Potential Contribution to High-Level Quinolone Resistance. Antibiotics.

[38] Kraupner N, Ebmeyer S, Bengtsson-Palme J, Fick J, Kristiansson E, Flach CF (2018). Selective concentration for ciprofloxacin resistance in Escherichia coli grown in complex aquatic bacterial biofilms. Environ Int.

[39] Dong Z, Wang J, Wang L, Zhu L, Wang J, Zhao X (2021). Distribution of quinolone and macrolide resistance genes and their co-occurrence with heavy metal resistance genes in vegetable soils with long-term application of manure. Environ Geochem Health.

[40] Palacios Farias SE (2019). "Frecuencia de Escherichia coli resistente a antibióticos aisladas del agua del río Piura, Perú en un tramo de la ciudad".

[41] Montero L, Irazabal J, Cardenas P, Graham JP, Trueba G (2021). Extended-Spectrum Beta-Lactamase Producing-Escherichia coli Isolated From Irrigation Waters and Produce in Ecuador. Front Microbiol.

