# Supplementary material for: Resistance to cephalosporins and quinolones in Escherichia coli isolated from irrigation water from the Rímac river in east Lima, Peru
Source: Rev Peru Med Exp Salud Publica. 2024 Jun 13;41(2):114–20. doi: 10.17843/rpmesp.2024.412.13246 (PMC11300684; doi:10.17843/rpmesp.2024.412.13246)
Supplement: Supplementary material. — Available in the electronic version of the RPMESP. [file rpmesp-41-02-13246-s001.docx]

# Material suplementario

| **Antibiotic resistance pattern** | | | | | |  |  |  |  | **Isolates N** | **Resistant to Antibiotics** |
| --- | --- | --- | --- | --- | --- | --- | --- | --- | --- | --- | --- |
| STX | NA | SAM | AMC | IMP | MRP | FEP | CAZ | FOX | ATM | 2 | 10 Antibiotics |
| STX | NA | CN | SAM | AMC | FOX | CTX | CZ | FF |  | 1 | 9 Antibiotics |
| NA | CN | SAM | FEP | AMC | ATM | CTX | CZ | FF |  | 1 | 9 Antibiotics |
| STX | NA | CAZ | FEP | AMC | ATM | CTX | CZ | FF |  | 1 | 9 Antibiotics |
| STX | LEV | CIP | NA | IMP | AMC | FOX |  |  |  | 1 | 7 Antibiotics |
| NA | FEP | AMC | FOX | ATM | CTX | CZ |  |  |  | 1 | 7 Antibiotics |
| STX | LEV | CIP | NA | SAM | FEP | AMC |  |  |  | 1 | 7 Antibiotics |
| STX | NA | CAZ | FEP | AMC | ATM | MRP |  |  |  | 1 | 7 Antibiotics |
| CIP | NA | CAZ | FEP | AMC | CTX | CZ |  |  |  | 1 | 7 Antibiotics |
| LEV | CIP | NA | CN | SAM | AMC | FF |  |  |  | 1 | 7 Antibiotics |
| STX | LEV | CIP | NA | CN | SAM |  |  |  |  | 1 | 6 Antibiotics |
| STX | CIP | NA | CN | SAM | FEP |  |  |  |  | 1 | 6 Antibiotics |
| STX | LEV | CIP | NA | AMC |  |  |  |  |  | 1 | 5 Antibiotics |
| STX | LEV | CIP | NA | SAM |  |  |  |  |  | 1 | 5 Antibiotics |
| STX | NA | SAM | AMC | FF |  |  |  |  |  | 1 | 5 Antibiotics |
| STX | LEV | CIP | NA |  |  |  |  |  |  | 2 | 4 Antibiotics |
| STX | CIP | AMC | FOX |  |  |  |  |  |  | 1 | 4 Antibiotics |
| STX | CIP | AMC | FOX |  |  |  |  |  |  | 1 | 4 Antibiotics |
| CIP | NA | SAM | AMC |  |  |  |  |  |  | 1 | 4 Antibiotics |
| CIP | NA | FEP | FF |  |  |  |  |  |  | 1 | 4 Antibiotics |
| STX | NA | SAM |  |  |  |  |  |  |  | 1 | 3 Antibiotics |
| NA | SAM | FEP |  |  |  |  |  |  |  | 1 | 3 Antibiotics |
| NA | ATM | CTX |  |  |  |  |  |  |  | 1 | 3 Antibiotics |
| CIP | NA | FEP |  |  |  |  |  |  |  | 1 | 3 Antibiotics |
| STX | NA | FEP |  |  |  |  |  |  |  | 1 | 3 Antibiotics |
| STX | NA | AMC |  |  |  |  |  |  |  | 1 | 3 Antibiotics |
| LEV | CIP | NA |  |  |  |  |  |  |  | 1 | 3 Antibiotics |
| NA | SAM | AMC |  |  |  |  |  |  |  | 1 | 3 Antibiotics |
| STX | NA |  |  |  |  |  |  |  |  | 9 | 2 Antibiotics |
| CIP | NA |  |  |  |  |  |  |  |  | 4 | 2 Antibiotics |
| STX | FEP |  |  |  |  |  |  |  |  | 1 | 2 Antibiotics |
| NA | CN |  |  |  |  |  |  |  |  | 1 | 2 Antibiotics |
| LEV | CIP |  |  |  |  |  |  |  |  | 1 | 2 Antibiotics |
| NA | SAM | |  |  |  |  |  |  |  | 1 | 2 Antibiotics |
| STX | FF |  |  |  |  |  |  |  |  | 1 | 2 Antibiotics |
| NA |  |  |  |  |  |  |  |  |  | 6 | 1 Antibiotics |
| SAM |  |  |  |  |  |  |  |  |  | 5 | 1 Antibiotics |
| STX |  |  |  |  |  |  |  |  |  | 3 | 1 Antibiotics |
| FEP |  |  |  |  |  |  |  |  |  | 3 | 1 Antibiotics |
| CZ |  |  |  |  |  |  |  |  |  | 1 | 1 Antibiotics |
| AMC |  |  |  |  |  |  |  |  |  | 1 | 1 Antibiotics |
| Sensitive to all antimicrobials | | | | | |  |  |  |  | 26 | 0 Antibiotics |

Antibiotic resistance patterns in *Escherichia coli* isolated in irrigation water. trimethoprim sulfamethoxazole: STX, levofloxacin: LEV, ciprofloxacin: CIP, nalidixic acid: NA, ampicillin sulbactam: SAM, ceftazidime: CAZ, cefepime: FEP, amoxicillin-clavulanic acid: AMC, cefoxitin: FOX, aztreonam: ATM, cefotaxime: CTX, cefazolin: CZ, meropenem: MEM, imipenem: IMP, Fosfomycin: FF, amikacin: AK, gentamicin: CN.
