# Supplementary material for: Resistance to cephalosporins and quinolones in Escherichia coli isolated from irrigation water from the Rímac river in east Lima, Peru
Source: Rev Peru Med Exp Salud Publica. 2024 Jun 13;41(2):114–20. doi: 10.17843/rpmesp.2024.412.13246 (PMC11300684; doi:10.17843/rpmesp.2024.412.13246)
Supplement: Translation

# Translation: Resistencia a cefalosporinas y quinolonas en *Escherichia coli* aisladas de agua de riego del río Rímac en Lima Este, Perú

**Authors:** Mónica Huamán Iturrizaga, Gina Salvador-Luján, Liliana Morales, Jeanne Alba Luna, Lino Velasquez Garcia, Julio Daniel Pacheco Perez, Maria J. Pons

**Affiliations:** 1 Laboratorio de Ecología Microbiana, Facultad de Ciencias Biológicas de Universidad Nacional Mayor de San Marcos, Lima, Perú.; 2 Oficina de Epidemiología y Salud Ambiental, Instituto Nacional Materno Perinatal, Lima Perú.; 3 Laboratorio de Microbiología, Hospital Militar Central “Luis Arias Schereiber”, Lima, Perú.; 4 Laboratorio de Microbiología Hospital Nacional Guillermo Almenara Irigoyen, Lima, Perú.; 5 Laboratorio de Genética Molecular y Bioquímica. Universidad Científica del Sur, Lima, Perú.

**Keywords:** Escherichia coli, resistencia a antibióticos, agua de riego, productoras de BLEE, E. coli diarreagénica

## Abstract

**Objetivos.:**

Evaluar la presencia y sensibilidad a los antimicrobianos de cepas de *Escherichia coli* aisladas de 24 muestras de agua de riego del río Rímac de Lima Este, Perú.

**Materiales y métodos.:**

Las cepas de *E. coli* fueron identificadas por PCR. La susceptibilidad a los antibióticos se procesaron por el método de difusión en disco. Los genes implicados en betalactamasas de espectro extendido (BLEE), quinolonas y virulencia se determinaron por PCR.

**Resultados.:**

Todas las muestras superaron los límites permisibles establecidos en las Normas de Calidad Ambiental para el riego de hortalizas. De los 94 aisaldos, el 72,3% mostró resistencia al menos a un antibiótico, el 24,5% eran multirresistentes (MDR) y el 2,1% extremadamente resistentes. Los mayores porcentajes de resistencia se observaron para ampicilina-sulbactam (57,1%), el ácido nalidíxico (50%), trimetoprim-sulfametoxazol (35,5%) y ciprofloxacino (20,4%). Entre los aislados, el 3,2% presentaba fenotipo BLEE relacionado con el gen *bla*_CTX-M-15_. Los mecanismos transferibles de resistencia a las quinolonas, *qnrB* fueron más frecuentes (20,4%), y el 2,04% tenían el *qnrS*. Se calcularon que el 5,3% eran *E. coli* diarreagénicas y de estas, el 60% eran *E. coli* enterotoxigénicas, el 20% *E. coli* enteropatógenas y el 20% *E. coli* enteroagregantes.

**Conclusiones.:**

Los resultados muestran la existencia de patotipos diarreogénicos en el agua utilizada para el riego de productos frescos y destaca la presencia de *E. coli* productores de BLEE y MDR, demostrando el papel que juega el agua de riego en la diseminación de genes de resistencia en el Perú.

## INTRODUCCIÓN

La aparición de bacterias resistentes a los antibióticos es una amenaza para la salud pública mundial, que necesita un enfoque multidisciplinario para integrar el conocimiento sobre “Una Salud” incluyendo el medio ambiente, los humanos y los animales ^1^. Los sistemas acuáticos han sido identificados como importantes reservorios de resistencia ^2^^,^^3^, proporcionando rutas de diseminación y transmisión para que las bacterias resistentes a los antimicrobianos se transfieran a humanos y animales ^4^.

La propagación de la resistencia a los antibióticos en los sistemas acuáticos merece especial atención considerando que el uso del agua puede facilitar la diseminación de bacterias a los humanos (por ejemplo, en uso de boca, riego, recreación y/o pesca) ^5^^-^^7^. Así mismo, en estos entornos, la presencia de otros compuestos, como metales y/o desinfectantes, se ha relacionado con la co-selección o selección de resistencias, que se acumula en los sistemas acuáticos contaminados ^8^^,^^9^. Además, se ha identificado en bacterias ambientales acuáticas el origen de algunos de los genes de resistencia a los antibióticos más extendidos a nivel mundial asociados con infecciones humanas (p. ej., *bla*_CTX -M_) ^10^.

En Perú, el agua del río Rímac pertenece a la cuenca fluvial más importante del país. Se estima que el 15% de sus recursos hídricos se utilizan en la agricultura, siendo la principal fuente de agua para las parcelas agrícolas del este de Lima ^11^. Los indicadores de contaminación fecal de este río exceden los límites de categoría para riego de vegetales (1000 número más probable [MPN] /100 ml) establecidos por los Estándares de Calidad del Agua del Ministerio del Ambiente en Perú ^11^.

Cabe señalar que no solo los microorganismos patógenos son relevantes en la movilización de mecanismos de resistencia, sino también bacterias comensales como *Escherichia coli*, la cual es considerada una de las especies más representativas de la microbiota del intestino tanto en humanos como en animales ^12^. La presencia de *E. coli* se utiliza como indicador de la calidad del agua, de los alimentos y algunos patotipos factores de virulencia asociados a diarrea (*E. coli diarreogenica*).

Dentro de los antimicrobianos, el grupo de las cefalosporinas y las quinolonas, se encuentran entre los más utilizados en humanos y animales de producción. Entre los mecanismos de resistencia más importantes en salud, y ampliamente distribuidos en la comunidad, son las betalactamasas de espectro extendido (BLEE), que confieren resistencia a los antibióticos betalactámicos, así como los mecanismos asociados a la resistencia a los aminoglucósidos y a las quinolonas, debidos principalmente a mutaciones cromosómicas, además de los mecanismos transferibles ^13^^,^^14^.

Comprender mejor la resistencia a los antibióticos en sistemas de riego específicos es esencial para crear estrategias de mitigación en la agricultura. En el Perú, la información sobre los niveles de resistencia en bacterias aisladas de cuerpos de agua es limitada, especialmente en agua de riego ^12^. Por consiguiente, el objetivo del presente estudio fue determinar los niveles de resistencia a los antibióticos y realizar la caracterización molecular de BLEE y mecanismos transferibles de resistencia a quinolonas en *E. coli* aislada de agua de riego de Lima Este, Perú.

**MENSAJES CLAVE**

Motivación para realizar el estudio. Los sistemas acuáticos, incluida el agua de riego, han sido identificados como reservorios de resistencia a antimicrobianos, siendo escasos los estudios en el Perú sobre la presencia de *Escherichia coli* y sus niveles de virulencia y resistencia antimicrobiana.

Principales hallazgos. El estudio reporta la presencia de bacterias *E. coli* por encima de la norma establecida para aguas de riego de vegetales, algunas con niveles muy altos de resistencia a los antimicrobianos.

Implicancias. La presencia de cepas productoras de BLEE de betalactamasas de espectro extendido y *E. coli* multirresistente, en el agua de riego podría contribuir en la diseminación de genes de resistencia en el Perú, siendo una amenaza importante en la salud pública.

## MATERIALES Y MÉTODOS

### Área de estudio

Se realizó un estudio observacional de corte transversal. Las muestras de agua se tomaron en 24 puntos de muestreo de agua de riego en 5 zonas de cultivo vegetal pertenecientes a los distritos de Lurigancho, Chaclacayo, Pachacamac, La Molina y Lurín, ubicados en la margen oriental del río Rímac al este de Lima Perú, entre octubre de 2019 y febrero de 2020 (Figura 1). Para el muestreo, se identificaron los campos agrícolas más grandes, tomando muestras de agua de los canales de riego que ingresaban a estas grandes extensiones parcelas principalmente con cultivos de verduras y hortalizas de tallo corto. Debemos mencionar que no se observaron cerca fuentes de agua potable ni había indicios de la presencia de ganado ovino, bovino o de otro tipo.

**Figura 1:** Mapa con puntos de muestreo de agua de riego en el este de Lima, Perú.

El estudio fue aprobado por el Vicerrectorado de Investigación y Postgrado de la Universidad Nacional Mayor de San Marcos bajo el código B19101681.

### Aislamiento e identificación de *Escherichia coli*

Las muestras fueron recolectadas siguiendo el protocolo RD160-2015-DIGESA y transportadas al laboratorio de Ecología Microbiana de la Facultad de Ciencias Biológicas de la Universidad Nacional Mayor de San Marcos ^15^. Las muestras se procesaron utilizando el método Colilert-18/Quanti-Tray para analizar coliformes totales y *E. coli* en todo tipo de agua (ISO 9308-2:2012). Se seleccionaron las cepas fenotípicamente sospechosas de ser *E. coli* y se almacenaron en agar tripticasa soja (TSA) a 8 °C para pruebas de susceptibilidad a antibióticos y a -80 °C en leche descremada para pruebas moleculares realizadas en el Laboratorio de Genética Molecular de la Universidad Científica del Sur.

Las *E. coli* fueron identificadas molecularmente mediante amplificación del gen *uidA*. La extracción de ADN se realizó mediante choque térmico durante 5 minutos a 100°C, seguido de centrifugación a 13.000 rpm durante 5 minutos. El ADN extraído se almacenó a -20 °C hasta su uso. Para la amplificación del gen *uidA,* que codifica la enzima 3-glucuronidasa como diana para la detección de *E. coli*, se utilizaron los cebadores uidA-R de 652 pb CCA TCA GCA CGT TAT CGA ATC CTT 61 82,6µM uidA-F de un amplicón de 652 pb ^16^.

### Sensibilidad a los antimicrobianos

Las cepas de *E. coli* confirmadas se reactivaron en agar TSA y se realizó el antibiograma con el método de difusión en disco de Kirby Bauer frente a 17 antibióticos: ácido nalidíxico (30 µg), trimetoprim sulfametoxazol (1. 25/75 µg), ciprofloxacino (5 µg), ampicilina-sulbactam (10/10 µg), cefepima (30 µg), amoxicilina-ácido clavulánico (20/10 µg), levofloxacino (5 µg), gentamicina (10 µg), fosfomicina (200 µg), aztreonam (30 µg), cefotaxima (30 µg), cefazolina (30 µg), cefoxitina (30 µg), ceftazidima (30 µg), imipenem (10 µg), meropenem (10 µg) y amikacina (30 µg). Los halos de inhibición se interpretaron siguiendo la directriz CLSI 2019 ^38^. Para la detección fenotípica de BLEE se utilizó el método Jarlier. Para las betalactamasas de tipo AmpC, se utilizó el método de sinergia de doble disco, y el cribado de carbapenemasas se consideró con halos de inhibición de imipenem y meropenem < 22 mm. Las cepas de control utilizadas fueron *E. coli* ATCC 25922 y ATCC 35218. Para obtener una visión general, las cepas con halos de inhibición intermedios y resistentes se incluyeron en la categoría resistente. La multirresistencia (MDR) se definió como la ausencia de sensibilidad adquirida a al menos un agente en tres o más categorías antimicrobianas, la extrema resistencia (XDR) se definió como la resistencia a tres o más familias de antimicrobianos, incluidos los carbapenémicos. ^1^^,^^17^^,^^18^.

### Patotipo diarreógeno de *Escherichia coli*

Se detecaron ocho genes de virulencia asociados con genes diarreógenos de *E. coli* (DEC) mediante PCR multiplex: enterotoxigénico (lt, st), enteropatógeno (eaeA), productor de toxina Shiga (stx1, stx2), *E. coli* enteroinvasiva (ipaH), enteroagregativa (aggR) y difusamente adherente (daaD) ^19^.

### BLEE y mecanismos de resistencia a quinolonas transferibles

Se realizó la caracterización molecular de genes BLEE de *E. coli* en cepas que presentaron el fenotipo BLEE*.* La amplificación se realizó por PCR para *bla*_CTX -M_, *bla*_TEM_ y *bla*_SHV._^20^.

La presencia de los genes *qnrA*, *qnrB*, *qnrC*, *qnrD*, *qnrVC*, *qnrS*, *qepA* y *oqxAB* se determinó mediante PCR en cepas con susceptibilidad disminuida (resistencia o intermedia) al ácido nalidíxico ^14^.

## RESULTADOS

### Aislamiento e identificación de *Escherichia coli*

Los recuentos totales de coliformes fecales y *E. coli* superaron los 2400 NMP/100 ml en las 24 muestras de agua procesadas. Entre las muestras positivas para Colilert, se aislaron 118 colonias sospechosas de *E. coli;* de ellas, 95 (79,2%) fueron confirmados mediante identificación molecular del gen *uidA*.

### Susceptibilidad a los antimicrobianos

Se encontraron importantes niveles de resistencia a ampicilina sulbactam (57,1%), ácido nalidíxico (50,0%), trimetoprim-sulfametoxazol (35,5%), amoxicilina-ácido clavulánico (22,0%) y ciprofloxacino (20,4%). Con relación a la resistencia a betalactámicos, la resistencia a cefepima fue del 18,9%, seguida de cefazolina y cefotaxima con un 6,3% de resistencia. El único antibiótico que no presentó resistencia fue la amikacina (0%) (Figura 2).

**Figura 2:** Niveles de resistencia a antibióticos en *Escherichia coli* aislada en agua de riego en Lima Este 2019-2020. Trimetoprima sulfametoxazol.

Las pruebas de susceptibilidad a los antimicrobianos encontraron que 68 (72,3%) cepas eran resistentes a al menos un antibiótico. Cabe destacar que 23 (24,5%) cepas eran MDR y 2 (2,1%) eran XDR. Finalmente, 26 (27,6%) cepas fueron sensibles a todos los antibióticos. (Material suplementario)

### Determinación del patotipo diarreógeno en *Escherichia coli*

De los 94 aislados *de E. coli*, 5 (5,3%) tenían al menos un gen de virulencia diarreógeno, y de estos, 3 (60%) tenían el gen de virulencia enterotoxigénico (*lt*, *st*) siendo ETEC, 1 (20,0%) enteropatógeno (*eaeA*) EPEC y 1 (20,0%) enteroagregativa (*aggR*) EAEC.

### Detección de ESBL y AmpC

En cuanto a los mecanismos de resistencia a los betalactámicos, se detectaron betalactamasas tipo AmpC fenotípicamente en 5 (5,3%) cepas y el fenotipo BLEE en 3 (3,2%) cepas. Además, todas las cepas productoras de BLEE tenían el gen *bla*_CTX-M-15_.

### Mecanismos transferibles de resistencia a las quinolonas (TMQR)

De las 49 cepas con sensibilidad disminuida a las quinolonas, 10 cepas presentaron el gen *qnrB* (20,4%), siendo la más frecuente, y sólo una cepa (2,0%) presentó el gen *qnrS*.

## DISCUSIÓN

La presencia de *E. coli* en ambientes acuáticos se ha relacionado con el vertido de aguas residuales, ya sean de uso doméstico o industrial, provocando la liberación al medio ambiente de bacterias resistentes a los antibióticos ^21^. Cabe señalar que los canales por donde fluyen las aguas agrícolas muestreadas se encontraban al aire libre, por lo que la contaminación pudo haber tenido diversos orígenes, tales como introducción de aguas residuales, vertidos domiciliarios y excrementos de hogares y/o animales salvajes ^21^^,^^22^.

El número de coliformes totales y *E. coli* en los 24 puntos de muestreo de agua de riego superó el límite permisible establecido por la normativa nacional (DS N°004-2017-MINAM para riego de hortalizas) ^23^, en concordancia con informes anteriores de organismos nacionales ^24^. La mala calidad del agua utilizada para riego es una de las razones de la presencia de patógenos en hortalizas de tallo corto, las cuales pueden contaminarse en cualquier etapa de la cadena alimentaria, desde la siembra hasta el consumidor ^25^. Además, la contaminación del agua de riego y la prevalencia de *E. coli* en hortalizas afecta la salud humana ^26^.

Además, se han detectado cepas de *E. coli* diarreogénicas (5,3%). Estas cepas se caracterizan por su capacidad de provocar patologías en animales y humanos por la transmisión de enfermedades transmitidas por alimentos, comprometiendo con ello el aprovechamiento del agua para el riego de hortalizas. Este porcentaje es menor en comparación con otros países de la región, como Chile, que reportó un 10% de cepas *de E. coli* diarreogénicas en aguas superficiales utilizadas en riego de hortalizas mediante un método de filtración tangencial, ^27^^)^ que tiende a concentrar la carga bacteriana, y 14% encontrado en agua de riego en Sinaloa, México. Nuestros hallazgos muestran que ETEC fue el patotipo aislado con mayor frecuencia, aunque también se han informado EPEC y EAEC. Estudios previos de *E. coli* diarreogénicas en una cohorte de niños de Lima han descrito estos patotipos, siendo ETEC más frecuente en niños de 2 a 12 meses de edad con diarrea ^28^. En estas cepas se encontraron altos niveles de resistencia a las sulfonamidas, y también a las quinolonas que no se utilizan habitualmente en este grupo de edad ^28^.

La presencia de resistencia a los antibióticos y los respectivos genes involucrados está generalmente vinculada al efecto antropogénico, como las heces humanas y las aguas residuales ^21^ y también se relaciona con una alta carga de contaminantes (metales pesados, antibióticos y pesticidas) en las aguas, debido principalmente a las actividades de la industria (minería), el crecimiento demográfico y las actividades agrícolas ^29^. Es importante destacar que, en nuestro estudio, el área muestreada no había canales de residuos hospitalarios o industriales ^29^.

En los últimos años, se ha informado de un aumento en los niveles de resistencia a los antimicrobianos en el agua de riego en Texas ^30^^)^ y también en la región de América Latina. ^31^. Así, en el presente estudio, el 72,3% de las cepas *de E. coli* eran resistentes a al menos un antibiótico, el 24,5% eran MDR y el 2,1% eran XDR. De hecho, los niveles de resistencia son extremadamente altos en la mayoría de los sistemas fluviales (hasta el 98% del total de bacterias detectadas), seguidos de los lagos, habiéndose informado valores más bajos en estanques y manantiales (<1%) ^32^.

Los fenotipos de resistencia más frecuentes en este estudio estaban relacionados con las quinolonas y las sulfonamidas. La presencia de genes relacionados con la resistencia a sulfonamidas se ha relacionado con efluentes de aguas residuales (no clorados ni declorados) ^33^. Por otro lado, la resistencia a quinolonas se ha incrementado en relación con el uso terapéutico y de promotores de crecimiento, que ha ido en aumento a nivel mundial ^34^. En Perú, al igual que en otras zonas de la región, se han reportado altos niveles de resistencia a quinolonas en microorganismos aislados tanto de niños diarreicos como sanos ^35^^-^^36^, lo que indica la alta presión de este antibiótico en la población.

En cuanto a los mecanismos transferibles implicados en la resistencia a las quinolonas, los medios acuáticos se han considerado importantes reservorios ^37^. Los estudios genéticos del agua han informado de la presencia de los genes *qepA* y *aac (6’)-Ib-cr*, que codifican la resistencia a las fluoroquinolonas, en un alto porcentaje de muestras de aguas residuales y lodos ^38^. En el presente estudio, el gen *qnrB* (20,4%) fue el mecanismo transferible de resistencia a las quinolonas (TMQR) más frecuente, y sólo un aislado presentó el gen *qnrS*. Anteriormente se estableció una correlación entre la presencia de un TMQR, como *qepA* y *qnrS*, y la cantidad de Cu y Zn en suelos vegetales con aplicación de estiércol a largo plazo, correlacionando los metales pesados con la persistencia de genes de resistencia a antibióticos ^39^_._

Nuestros hallazgos de cepas BLEE (3,2%) fueron menores comparados con los reportados por Palacios (16,1%) en agua del río Piura, Perú ^34^, y el 29% de BLEE en *E. coli* de agua de riego de Ecuador ^40^. Sin embargo, estos resultados no son comparables porque, en estos estudios, se seleccionaron con medios que contenían antibióticos de la familia de las cefalosporinas. Asimismo, se detectó el gen *bla*_CTX-M-15_, que junto con los genes *bla*_CTX-M-55_, *bla*_CTX-M-65_ fueron los genes encontrados más frecuentemente en asociación con el fenotipo BLEE, y también fueron los alelos más frecuentes asociados con infecciones humanas ^3^^,^^14^^,^^41^.

El estudio presenta algunas limitaciones relacionadas con el tamaño de las muestras tomadas, teniendo en cuenta la gran extensión de la zona. Además, no se utilizaron medios de cultivo de cribado para la detección de cepas BLEE, lo que habría ayudado a aislar un mayor número de *E. coli* resistentes a este grupo de antimicrobianos. A pesar de ello, la importancia de este estudio radica en la aportación de nuestros hallazgos en la escasa información de investigaciones previas en *E. coli* entre aguas de riego con resistencia a diferentes familias de antibióticos (quinolonas, aminoglucósidos, betalactámicos, monobactámicos, sulfonamidas). Asimismo, el hallazgo de patotipos de *E. coli* hace evidente la necesidad de mejorar las políticas y el control del agua de riego en las diferentes zonas agrícolas de Lima. Esto es particularmente importante en Perú, que presenta una baja frecuencia de tratamiento de aguas residuales.

En conclusión, este estudio pone de manifiesto la presencia de coliformes fecales por encima del límite permisible establecido por la norma nacional. Además, demuestra la existencia de *E. coli* diarreagénicos y altos niveles de resistencia a quinolonas y sulfonamidas, con especial preocupación a *E. coli* productores de BLEE, en las aguas de riego de la periferia de Lima, representando un peligro potencial para la salud de animales y humanos.
